# GRHL3/GET1 and Trithorax Group Members Collaborate to Activate the Epidermal Progenitor Differentiation Program

**DOI:** 10.1371/journal.pgen.1002829

**Published:** 2012-07-19

**Authors:** Amelia Soto Hopkin, William Gordon, Rachel Herndon Klein, Francisco Espitia, Kenneth Daily, Michael Zeller, Pierre Baldi, Bogi Andersen

**Affiliations:** 1 Department of Biological Chemistry, University of California Irvine, Irvine, California, United States of America; 2 Department of Medicine, University of California Irvine, Irvine, California, United States of America; 3 Center for Complex Biological Systems, University of California Irvine, Irvine, California, United States of America; 4 Department of Computer Science, University of California Irvine, Irvine, California, United States of America; Medical Research Council Human Genetics Unit, United Kingdom

## Abstract

The antagonistic actions of Polycomb and Trithorax are responsible for proper cell fate determination in mammalian tissues. In the epidermis, a self-renewing epithelium, previous work has shown that release from Polycomb repression only partially explains differentiation gene activation. We now show that Trithorax is also a key regulator of epidermal differentiation, not only through activation of genes repressed by Polycomb in progenitor cells, but also through activation of genes independent of regulation by Polycomb. The differentiation associated transcription factor GRHL3/GET1 recruits the ubiquitously expressed Trithorax complex to a subset of differentiation genes.

## Author Summary

Human epidermal keratinocyte differentiation provides a highly suitable system to understand how progenitor cells become specialized. Previous work has implicated resolution of repressive histone modifications in the activation of the terminal differentiation gene expression program. Our work shows that this mechanism only accounts for the regulation of a subset of the differentiation gene expression program and that activating histone modifications by Trithorax chromatin modifiers, acting alone or in combination with the release from repressive chromatin changes, is essential. Furthermore, we show that the Trithorax complex is recruited to a subset of differentiation gene promoters by the transcription factor Grhl3, an evolutionarily conserved regulator of the epidermal differentiation program. Altered differentiation is characteristic for several skin diseases, including skin cancer and inflammatory diseases such as psoriasis. While genetic abnormalities play a role in these diseases, the cellular and macro-environment may also alter the course of these diseases through chromatin changes (epigenetics). Understanding the epigenetic regulation of keratinocyte differentiation may in the future lead to the development of new drugs for skin diseases.

## Introduction

Epigenetic control of cell fate by the opposing action of the repressive Polycomb group proteins (PcG) and the activating Trithorax group proteins (trxG) is a mechanism found throughout evolution [1]–[3]. These families of chromatin modifiers were first described as regulators of HOX gene expression in *Drosophila* [4], [5]. The mammalian counterparts of PcG and trxG have since been identified and their role in the regulation of multiple cellular processes, outside of HOX gene regulation, has begun to emerge [1]. Although trxG has been depicted as a de-repressor of PcG repressed genes in *Drosophila*, it remains unclear if in mammalian differentiation trxG mediated gene regulation is only through antagonizing PcG mediated gene repression, or if trxG can regulate gene activation independent of PcG [1].

The epidermis, the outermost skin layer, is a multi-layered epithelium containing proliferative progenitors at the base that migrate towards the surface while simultaneously undergoing differentiation to form an effective barrier which prevents dehydration and protects the organism against toxins and invasion of microorganisms [6], [7]. Epidermal differentiation involves the coordinated expression of numerous genes including those involved in protein cross-linking, lipid metabolism, and cell adhesion. As such this system represents an excellent model for dissecting the transcriptional and regulatory changes required for differentiation. Some key transcription factors regulating this process have been identified [8], including GRHL3/GET1 [9], [10], and more recently the contribution of epigenetic regulation has begun to emerge. DNA methylation [11] and histone deacetylation [12], as well as remodeling of chromatin by BRG1 [13] and Mi-2beta [14], have been described in regulating various stages of epidermal differentiation and homeostasis. Furthermore, the chromatin organizer Satb1, shown to be directly regulated by the transcription factor p63, regulates the expression of certain epidermal differentiation associated genes [15]. Epidermis-specific deletion of Ezh2, a PcG histone methyltransferase of the polycomb repressive complex 2 (PRC2) that catalyzes the methylation of Lys27 on histone 3 (H3K27), led to the premature expression of some but not all late differentiation genes [16]. Consistently, it was recently reported that epidermal deletion of the PRC2 related protein JARID2 leads to a loss of H3K27me3 at many of the Ezh2 affected epidermal differentiation genes [17]. Moreover, the PRC1 related protein Cbx4 was shown to maintain human epidermal stem cells in the proliferative state while also preventing them from senescence [18]. Complementing these findings, JMJD3, a histone demethylase that removes the repressive H3K27me3, promotes epidermal differentiation [19]. Thus, there is abundant evidence indicating that PcG-mediated H3K27 methylation maintains keratinocytes in the progenitor state and release from this repression contributes to epidermal differentiation. Yet, addition and removal of the H3K27me3 mark does not fully explain differentiation associated gene activation as many differentiation genes are not affected by interference with either Ezh2 or JMJD3 [16], [19]. We now show that the trxG components, histone H3K4 methyltransferase MLL2 and WDR5, play an important role in epidermal differentiation and, in part, act to regulate gene expression independent of PcG. Furthermore we demonstrate that a subset of epidermal differentiation genes are activated by GRHL3 mediated recruitment of trxG.

## Results

### Differentiation related increase in H3K4 methylation at the *TGM1* promoter depends on GRHL3

**Figure 1:**
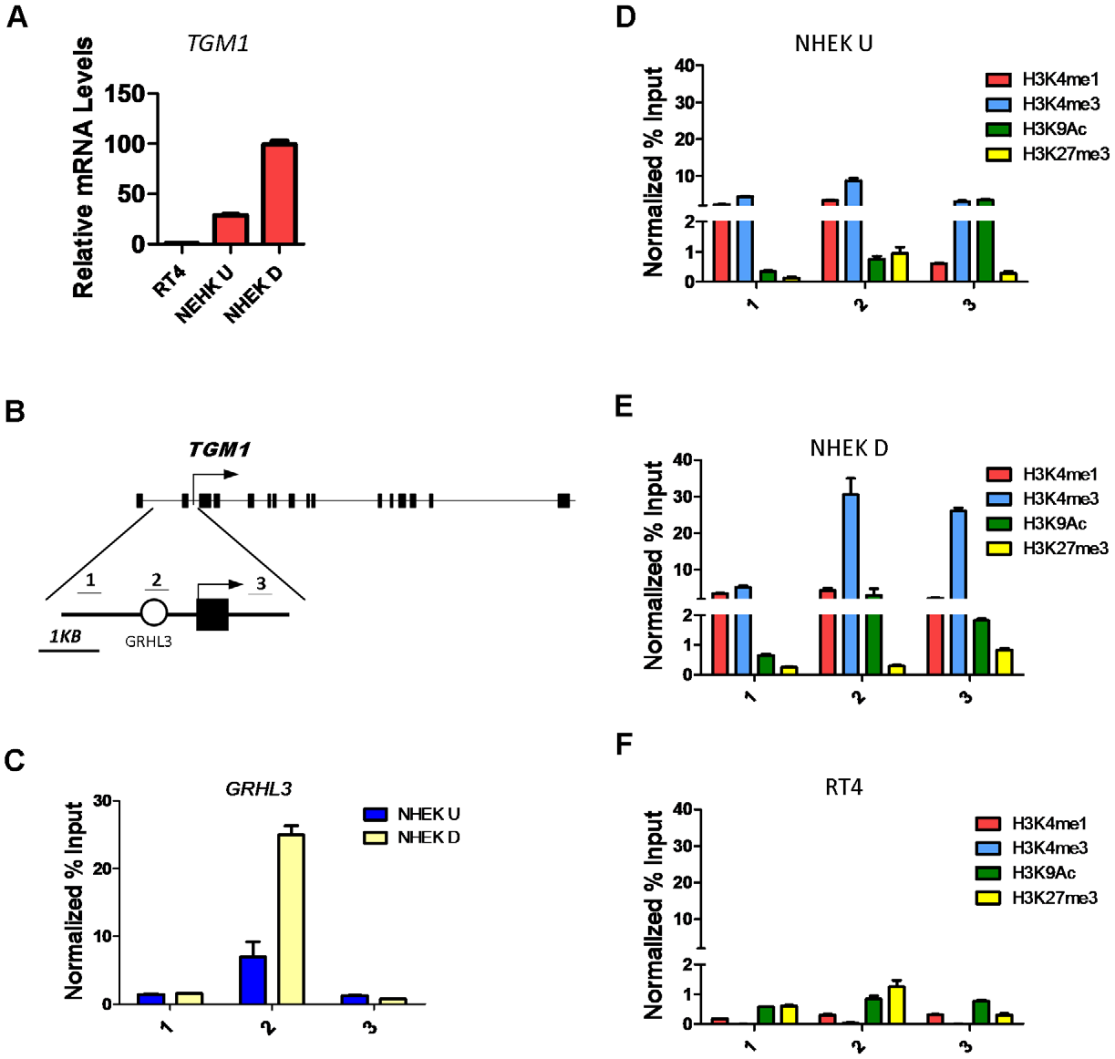
Histone landscape at the *TGM1* promoter. (A) qRT-PCR of *TGM1* expression in human bladder epithelia cells (RT4), undifferentiated normal human epidermal keratinocytes (NHEK U), and differentiated normal human epidermal keratinocytes (NHEK D). (B) Schematic of the human *TGM1* gene. Boxes denote exons, circle denotes predicted GRHL3 binding site, arrow denotes transcription start site, and numbers correspond to location of primer pairs. (C) GRHL3 ChIP assays in NHEK U and NHEK D cells. Numbers below the X-axis denote primers specified in B. (D–F) ChIP assays for Histone 3 Lysine 4 (H3K4) mono (1), and tri (3) methylation, H3K9 acetylation (Ac) and H3K27me3 in (D) NHEK U, (E) NHEK D, and (F) RT4 cells. Numbers below the X-axes denote primers specified in B. For ChIP assays data is mean and error bars represent SD; for mRNA expression data is mean and error bars represent SEM.

The highly conserved Grainyhead transcription factors control epidermal differentiation and barrier formation in organisms ranging from worm to human by directly or indirectly regulating the expression of key genes involved in these processes [9], [10], [20]–[22]. One Grainyhead homologue, the mouse Grhl3/Get1, is a critical regulator of the epidermal differentiation program [9], [22]. To study GRHL3 gene-regulatory mechanisms we utilized the *in vitro* calcium induced differentiation model of normal neonatal human epidermal keratinocytes (NHEK). In this system *GRHL3* is expressed at low levels in undifferentiated cells and at higher levels when cells are induced to differentiate (Figure S1A), reminiscent of its high expression in the most differentiated layer of mouse skin. Likewise the GRHL3 target Transglutaminase 1 (*TGM1*), a Ca^2+^-dependent enzyme that functions in the formation of the cornified cell envelope by crosslinking proteins such as Involucrin and Filaggrin, is increased 3-fold upon calcium-induced differentiation (Figure 1A). The human *TGM1* promoter contains a conserved GRHL3 binding site ∼800 bp upstream of the transcription start site (TSS) (Figure 1B) within a 2.5 Kb region that mediates correct temporal and spatial expression in transgenic mice [23]. To determine if *TGM1* is a direct target of GRHL3 in NHEKs, we performed chromatin immunoprecipitation (ChIP) assays with a GRHL3 antibody in undifferentiated and differentiated NHEKs with three primer pairs tiling the *TGM1* promoter (Figure 1B). Consistent with a differentiation-dependent increase in *TGM1* expression, we observed a differentiation-dependent increase in GRHL3 occupancy at the predicted binding site in the *TGM1* promoter (Figure 1C); this binding was specific as no binding was detected upstream or downstream (Figure 1C). Higher occupancy of GRHL3 on the *TGM1* promoter in differentiated NHEKs correlated with increased H3K4me3, a histone modification associated with active promoters (Figure 1D, 1E). Additionally we found low levels of H3K27me3 in differentiated NHEKs compared to slightly higher levels in undifferentiated NHEKs and RT4 cells, a human bladder epithelial cell line which expresses *GRHL3* but virtually no *TGM1* (Figure 1A and 1D–1F, Figure S1A). RT4 cells also displayed very low levels of the H3K4me1 and H3K4me3 modifications consistent with the low level of *TGM1* expression (Figure 1F). These experiments illustrate a distinct chromatin landscape in *TGM1*-expressing and non-expressing cell types and increased H3K4 methylation at the *TGM1* promoter correlating with increased GRHL3 binding and *TGM1* expression during differentiation.

**Figure 2:**
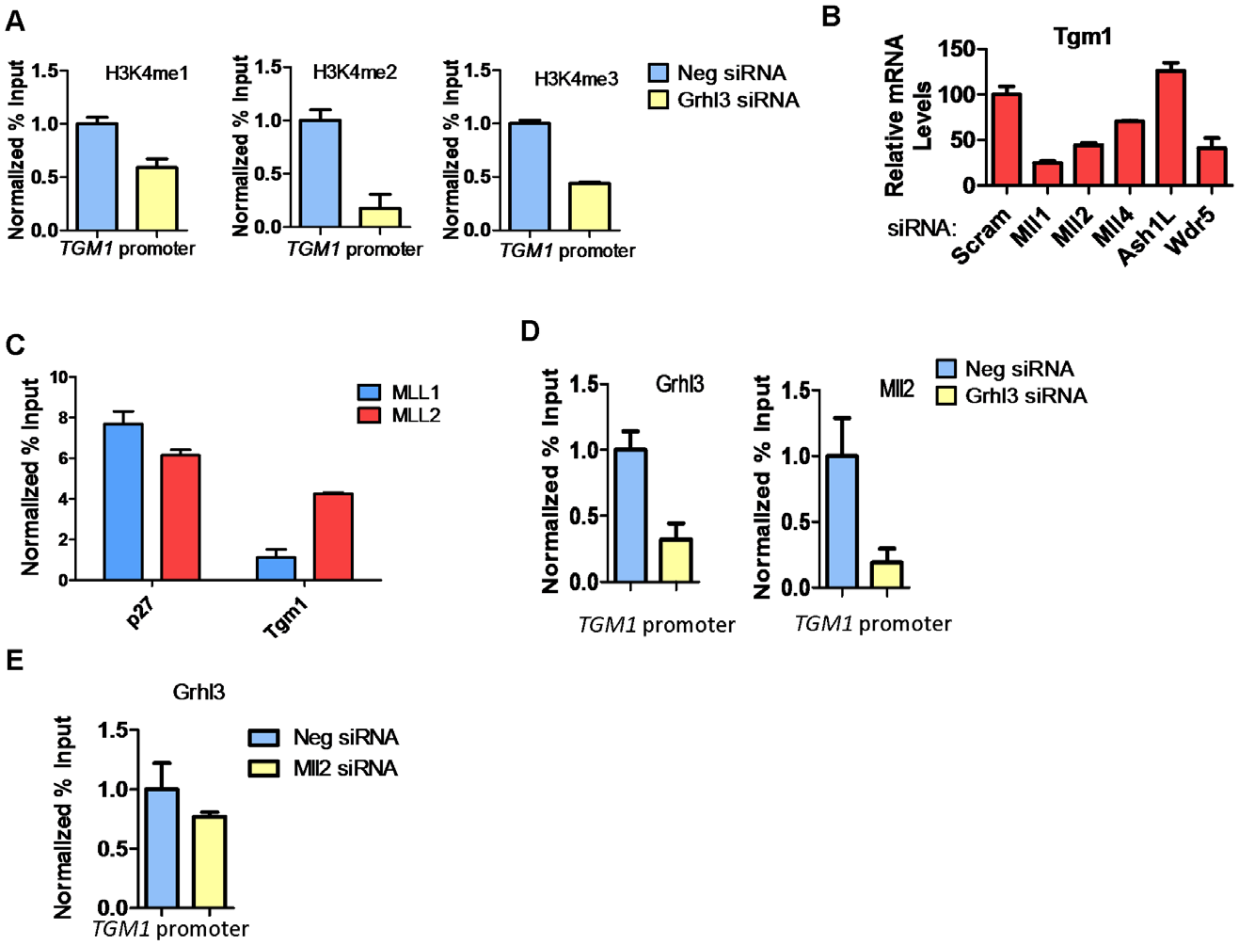
MLL2 occupancy and H3K4 methylation at the *TGM1* promoter depend on GRHL3 in human epidermal keratinocytes. (A) ChIP assays for H3K4 -me1, -me2, -me3, at the *TGM1* promoter in NHEK D treated with GRHL3 siRNA and Neg siRNA. (B)*TGM1* expression in NHEK D cells after treatment with indicated siRNAs. (C) ChIP assays with MLL1 and MLL2 antibodies in NHEK D cells, detected by q-PCR with primers to the *p27* and *TGM1* promoters. *p27* is used as a positive control for MLL1 ChIP. (D) ChIP assays with GRHL3 and MLL2 antibodies in Neg siRNA and GRHL3 siRNA treated NHEK D cells, detected by q-PCR with primers to the *TGM1* promoter region. (E) ChIP assay for GRHL3 in Neg siRNA and MLL2 siRNA treated NHEK D cells, detected by q-PCR with primers to the *TGM1* promoter region. For ChIP assays data is the mean normalized to Neg siRNA and error bars represent SD; for mRNA expression data is mean and error bars represent SEM.

To determine whether increased H3K4 methylation at the *TGM1* promoter depends on GRHL3 binding, we utilized siRNAs to knock down GRHL3 in NHEK cells followed by calcium-induced differentiation for 48 hours. As expected there is a greater than 2-fold reduction in *TGM1* mRNA levels upon knockdown of GRHL3 (Figure S1B). Correspondingly, we observed a strong decrease in H3K4 mono-, di-, and tri-methylation at the *TGM1* promoter in GRHL3 depleted cells (Figure 2A). These results indicate that increased H3K4 methylation at the *TGM1* promoter during differentiation of NHEK cells is facilitated by GRHL3.

As H3K4 methylation is mediated by the SET domain-containing trxG components, we examined the expression of these and core trxG complex members in NHEKs; all are expressed at a relatively stable level during human keratinocyte differentiation (Figure S2A). We also assessed expression of *GRHL3*, *MLL1*, *MLL2*, and *WDR5* transcripts by qPCR in human skin (Figure S2B) and mouse skin separated into dermal and epidermal compartments (Figure S2C). Transcripts encoding all four factors are easily detected in human skin with WDR5 and MLL1 having the highest expression (Figure S2B). In mouse skin that was separated into epidermis and dermis, we found that *Grhl3* and *Mll2* were enriched in the epidermis while both *Mll1* and *Wdr5* are expressed at a similar level in the epidermis and dermis (Figure S2C). We also assessed the expression of these same proteins in human skin by immunofluorescence and observed expression of MLL1, MLL2, and WDR5 throughout the epidermis with MLL2 and WDR5 showing higher expression in the more differentiated cells and MLL1 expression being absent from the nucleus of basal cells (Figure S2D–S2H).

Upon siRNA knockdown of individual trxG members followed by differentiation, there were varying effects on the expression of *TGM1*. Knockdown of MLL1 and MLL2 (also known as KMT2B and ALR) caused a greater than 2 fold reduction in *TGM1* expression, knockdown of MLL4 caused less of a reduction, and knockdown of ASHL1 caused a slight increase in *TGM1* expression (Figure 2B, Figure S2I). *TGM1* mRNA expression was also decreased upon knockdown of WDR5, a non-enzymatic core component of the methyltransferase complex, further supporting the role of trxG dependent histone methylation in *TGM1* expression (Figure 2B). ChIP assays revealed that MLL2 but not MLL1 occupied the *TGM1* promoter in differentiated NHEK cells (Figure 2C). MLL2 recruitment to *TGM1* depends on GRHL3 as there is a significant reduction in both GRHL3 and MLL2 at the *TGM1* promoter in GRHL3 depleted cells (Figure 2D). Conversely, when MLL2 was knocked down, GRHL3 recruitment to the *TGM1* promoter was not significantly affected (Figure 2E). Together these findings indicate that *TGM1* is a direct target of MLL2-mediated H3K4 methylation in NHEKs, and that this recruitment is GRHL3-dependent.

### GRHL3 and MLL2 co-regulate epidermal differentiation genes

**Figure 3:**
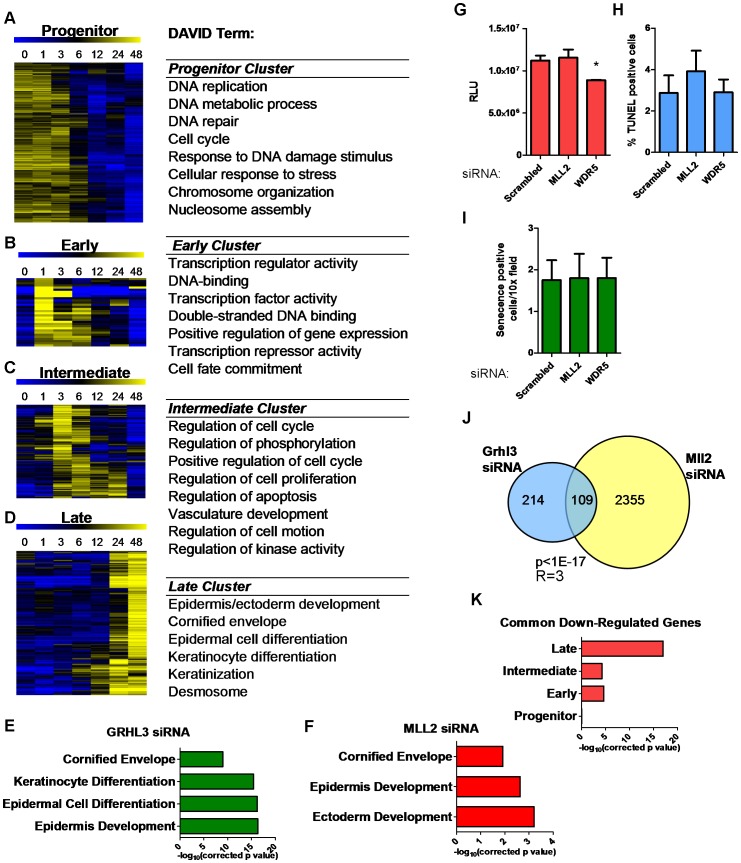
Loss of GRHL3 and MLL2 affects epidermal differentiation genes as defined by expression profiling during keratinocyte differentiation. (A–D) Heatmaps of K-means clusters and corresponding GO categories in (A) Progenitor, (B) Early, (C) Intermediate and (D) Late clusters. (E–F) GO categories of genes significantly downregulated after (E) GRHL3 siRNA and (F) MLL2 siRNA. (G) Proliferation assay in NHEK U cells treated with Neg, MLL2 and WDR5 siRNA. (H) TUNEL assay in NEHK U cells treated with Neg, MLL2, and WDR5 siRNA. (I) Senecence assay in NHEK U cells treated with Neg, MLL2 and WDR5 siRNA. (J) Venn diagram illustrating overlap between genes significantly downregulated by GRHL3 and MLL2 siRNAs. (K) Significance for the overlap of genes belonging to differentiation clusters in A–D and genes down-regulated by both GRHL3 siRNA and MLL2 siRNA. p values calculated by Fisher's exact test. R = enrichment factor.

To better understand the broader roles of GRHL3 and MLL2 in keratinocyte differentiation we utilized microarrays to define the global differentiation gene expression program in NHEKs at various time points during differentiation; 2,583 genes are significantly differentially expressed (p<0.005, fold-change>1.25) (Figure S3A, Table S1). The most overrepresented Gene Ontology (GO) terms included, “epidermis development”, “regulation of cellular proliferation”, “regulation of cell motion”, “cornified envelope”, and “positive regulation of gene expression” (Figure S3B). K-Means clustering of these genes revealed four clear patterns (Figure 3A–3D). First, a “progenitor” cluster; genes most highly expressed in undifferentiated cells with falling expression during the time course (Figure 3A), including E2F3 and CDC6, both of which have been linked to differentiation induced cell cycle arrest in keratinocytes [24], [25]. Second, an “early” cluster; genes expressed at low levels in undifferentiated cells that were up-regulated most highly at one hour of differentiation (Figure 3B, Figure S4), including many key transcription factors like Jun and Fos, AP1 components that play an important role in epidermal differentiation [26]. Furthermore, this cluster contains KLF4 and HES1, both of which have been shown to play a role in the induction of terminal differentiation [27], [28]. Third, an “intermediate” cluster; genes up-regulated most highly at three to six hours post induction with many genes related to kinase activity, including SRF [29], and positive apoptosis regulators including SOCS3 [30] (Figure 3C, Figure S4). Fourth, a “late” cluster; genes most highly upregulated at 24 and 48 hours, at the end of the time course with an overrepresentation of genes related to epithelial differentiation, keratinization, desmosomes, and the cornified layer including KRT1, KRT10, and FLG, as well as members of the Epidermal Differentiation Complex (Figure 3D, Figure S3). This cluster also contains CDKN2A (Ink4a) a powerful inhibitor of cell cycle progression shown to play a role in inhibiting G1 to S transition in the epidermis, which is critical for epidermal terminal differentiation [31]. In summary, through gene expression profiling over a dense time course of NHEK differentiation, we are able to recapitulate key aspects of normal epidermis differentiation and classify novel genes in this process.

To further investigate the hypothesis that GRHL3 recruits MLL2 to target promoters to activate gene transcription during epidermal differentiation we studied the effect of loss of GRHL3 and MLL2 on global gene expression in differentiated NHEK cells. We found 323 and 4,281 differentially expressed genes (p<0.001) when we depleted GRHL3 and MLL2, respectively, indicating a more general role for MLL2 than GRHL3 in keratinocyte transcription (Figure S5A–S5B and Tables S2, S3). DAVID analysis of down regulated genes revealed over-represented GO terms such as “cornified envelope” and “epidermal differentiation” in the GRHL3 siRNA dataset, indicating that GRHL3 plays a similar differentiation-promoting role in human keratinocytes as in mice (Figure 3E). Down regulated genes in the MLL2 siRNA experiment were also enriched for “cornified envelope” and “epidermis development” supporting the idea that MLL2 plays a crucial role in epidermal keratinocyte differentiation (Figure 3F). Knockdown of MLL2 in undifferentiated NHEKs had no effect on proliferation, apoptosis or senescence while knockdown of the core component WDR5 lead to a slight but significant decrease in proliferation and no change in apoptosis or senescence (Figure 3G–3I). Comparing the lists of significantly down-regulated genes in each data set, we found a statistically significant overlap (Figure 3J), and using MotifMap [32], we detected a statistically significant over-representation of predicted GRHL3 binding sites in the down-regulated genes (Figure S5C), providing additional evidence that GRHL3 and MLL2 co-occupancy regulates some of these genes.

The GRHL3 regulated geneset overlapped significantly with both the early and intermediate clusters (p<0.001) from our time course study of NHEK differentiation. The intersection with the late cluster was even more significant (p<1×10^−17^), fitting with the idea that, as in mouse development, GRHL3 is an important regulator of terminal differentiation in human keratinocytes (Figure S5D). When the genes affected by MLL2 siRNA were overlapped with the same four clusters, the most significant enrichment was found with both the intermediate and late clusters although there was a significant overlap with all four clusters, suggesting a broader role for MLL2 in the differentiation process (Figure S5E). Further exploration of the differentially expressed genes in common between the GRHL3 and MLL2 siRNA experiments showed the strongest overlap with the late cluster of genes, indicating that these two factors converge on terminal differentiation genes (Figure 3K). These findings support our hypothesis that GRHL3 acts, in part, to regulate gene expression through collaboration with MLL2 and that MLL2 itself is a crucial regulator of epidermal differentiation.

### GRHL3, MLL2, and MLL1 occupy multiple epidermal differentiation gene promoters

**Figure 4:**
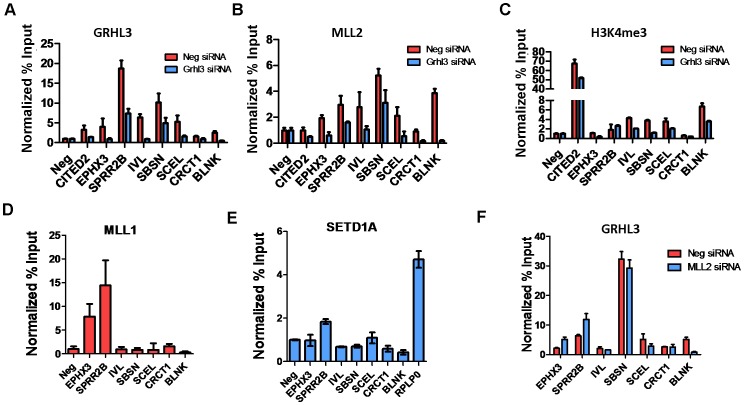
MLL2, MLL1, and GRHL3 co-occupy human epidermal keratinocyte differentiation genes. (A–C) ChIP assays with GRHL3 (A), MLL2 (B), and H3K4me3 (C) antibodies in Neg siRNA and GRHL3 siRNA treated NHEK D cells. ChIP assays with (D) MLL1 and (E) SETD1A antibodies in NHEK D cells. (F) ChIP assays with GRHL3 antibody in Neg siRNA and MLL2 siRNA treated NHEK D cells. Primer pairs amplify a region in the promoter of each gene where a predicted GRHL3 binding site is located.

To test whether GRHL3 and MLL2 co-occupy the promoters of epidermal differentiation genes other than *TGM1*, we identified a set of genes with the following criteria: 1) down regulated by siRNAs against both factors; 2) GO terms related to epidermal differentiation; and 3) containing a high scoring GRHL3 binding site within a −2 to +1 Kb region around the transcription start site. These putative GRHL3/MLL2 target genes were then tested for the presence of GRHL3, MLL2 and H3K4me3 by ChIP assays on chromatin from either scrambled or GRHL3 siRNA treated differentiated NHEKs. Consistent with our hypothesis many of the tested target genes were in fact bound by GRHL3 and MLL2 at their proximal promoters and displayed reduced H3K4me3 levels in GRHL3 siRNA treated cells, with the exception of SPRR2B which had no change in H3K4me3 (Figure 4A–4C). We also performed ChIP assays for MLL1 and SET1 occupancy and found that SPRR2B is also a target of both MLL1 and SET1 while EPHX3 is also a target of MLL1 (Figure 4D–4E). We also examined GRHL3 occupancy at these genes in cells depleted of MLL2 and found that with the exception of BLNK, GRHL3 occupancy was not significantly decreased, further supporting the hypothesis that GRHL3 contributes to MLL2 recruitment to target gene promoters and not vice versa (Figure 4F).

**Figure 5:**
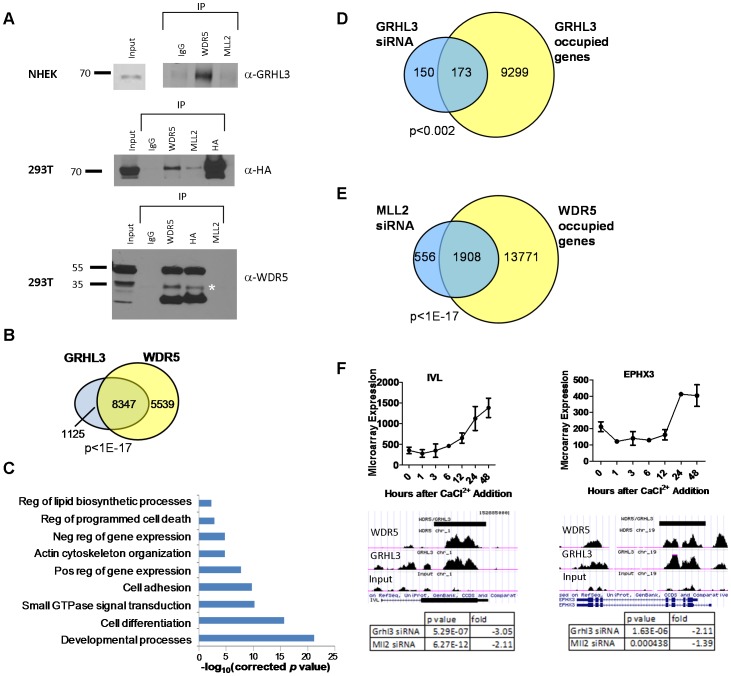
WDR5 and GRHL3 co-occupy human epidermal keratinocyte differentiation genes. (A) Co-Immunoprecipitation in NHEK D cells (top) and HA-GRHL3 transfected 293T cells (lower panels). Cell extracts were immunoprecipitated with the indicated antibodies and blots were probed with the indicated antibodies (IgG was used as a control). * denotes predicted WDR5 band. (B) Venn diagram illustrating number of genes with overlapping WDR5 and GRHL3 ChIP-seq peaks. (C) GO categories of the 8,347 genes with overlapping WDR5 and GRHL3 ChIP-seq peaks. (D–E) Venn diagrams illustrating overlap between genes significantly downregulated upon (D) GRHL3 siRNA mediated depletion and genes occupied by GRHL3, and genes downregulated upon (E) MLL2 siRNA mediated depletion and genes occupied by WDR5 (p values calculated by Fisher's exact test). (F) Examples of WDR5 and GRHL3 co-occupied genes, showing mRNA expression during the epidermal keratinocyte differentiation time course (top panels), and tables displaying respective gene expression changes upon depletion of GRHL3 and MLL2 by siRNA (bottom panels).

In order to test whether GRHL3 could directly bind trxG complex members, we performed Co-IP experiments on extracts from differentiated NHEK cells, and from 293T cells transfected with HA-GRHL3. While we could not detect GRHL3 in MLL2 immunoprecipitates in NHEK cells, we readily detected GRHL3 in extracts precipitated with WDR5, a core component of the trxG methyltransferase complex (Figure 5A). HA-GRHL3 was detected in both MLL2 and WDR5 immunoprecipitates in 293T cells, while WDR5, but not MLL1 or SET1, was readily detected in 293T extracts precipitated with HA antibody (Figure 5A, Figure S6A–S6B). Thus, GRHL3 appears to interact strongly with WDR5 and to a lesser degree with MLL2, consistent with the idea that GRHL3 can recruit trxG to target promoters.

### WDR5 and GRHL3 co-occupy epidermal differentiation genes

The above results led us to perform ChIP-seq experiments to define genome-wide GRHL3 and WDR5 co-occupancy in differentiated NHEK cells, identifying 25,340 GRHL3 peaks and 48,269 WDR5 peaks (FDR<5%) (Figure S6C). For both proteins there is a statistically significant enrichment in occupancy at promoters compared to the average genomic distribution (Figure S6D). Strikingly, we found that 88 percent of genes that contained a GRHL3 peak also had an overlapping WDR5 peak, further supporting the hypothesis that GRHL3 recruits WDR5 to gene regulatory regions (Figure 5B). GO analysis of these co-occupied targets revealed enrichment for processes like “cell differentiation”, “positive regulation of gene expression”, “regulation of programmed cell death”, “cell-cell adhesion”, and “regulation of lipid biosynthetic processes”, all important components of epidermal keratinocyte differentiation (Figure 5C). Forty three percent of genes differentially expressed during keratinocyte differentiation had co-occupancy of WDR5 and GRHL3 (Figure S6E), and there was a relatively even distribution of WDR5, GRHL3, and WDR5/GRHL3 co-occupied genes in our four differentiation clusters (Figure S6F). There is also a statistically significant overlap of genes bound by GRHL3 that were downregulated upon GRHL3 depletion by siRNA, as well as between genes bound by WDR5 and downregulated upon MLL2 depletion, supporting the idea that many of these genes are direct targets of GRHL3 and MLL2, respectively (Figure 5D–5F). Together these findings indicate a role for trxG in human epidermal keratinocyte differentiation and a novel role for GRHL3 in recruiting this complex to gene promoters.

### Epidermal keratinocyte differentiation is regulated by a combination of PcG and trxG or PcG-independent trxG-mediated regulation

**Figure 6:**
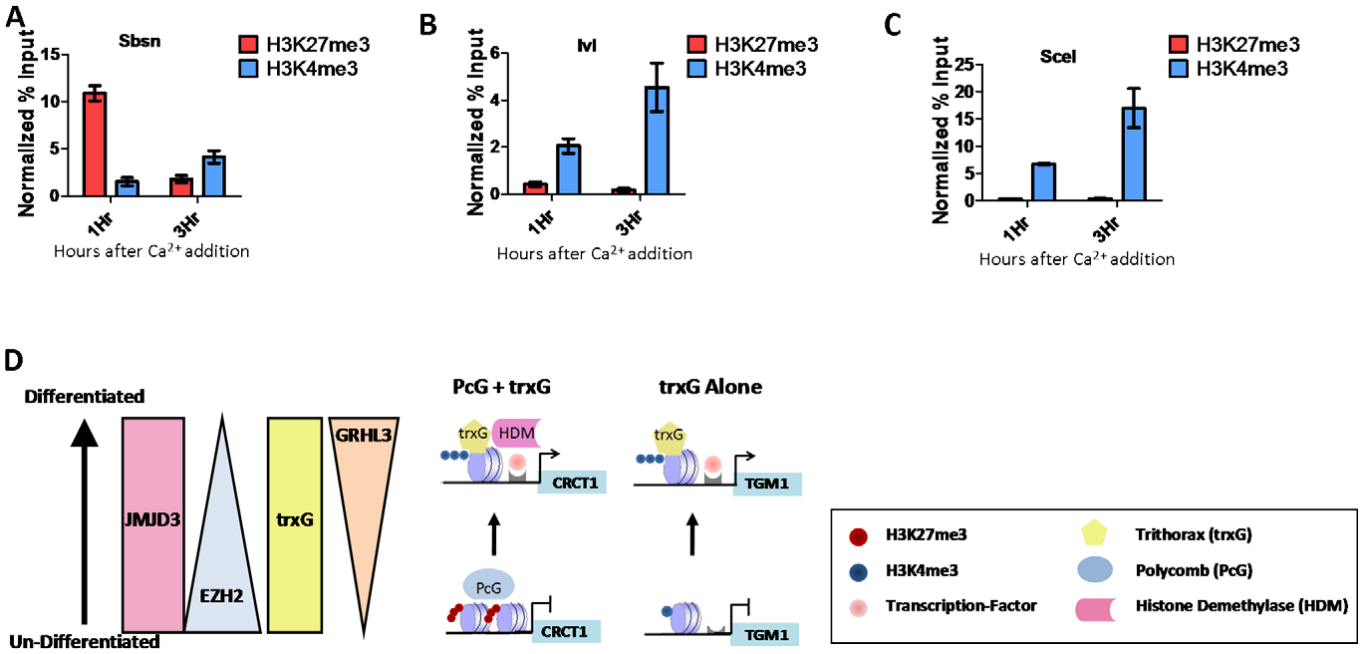
PcG and trxG regulation in epidermal differentiation. (A–C) ChIP assay with H3K27me3 and H3K4me3 antibodies 1 and 3 hours post calcium-induced differentiation in NHEK D cells. (D) Model of epidermal keratinocyte differentiation mediated by either PcG and trxG, or trxG alone.

To understand the relationship between trxG and PcG in differentiation and to determine if there are genes that are regulated by trxG independent of PcG, we performed ChIP assays at one and three hours after calcium-induction to examine the dynamics of H3K4me3 and H3K27me3 at the differentiation associated genes we had identified as common targets of GRHL3 and MLL2. Interestingly, different sets of genes showed unique dynamics of these marks, one group, including the genes CRCT1, SBSN, and EPHX3 showed initially high levels of H3K27me3 followed by a drastic decrease, coupled with only a modest increase in H3K4me3 (Figure 6A, Figure S7A). A predominant mechanism of PRC recruitment, described in human ES cells, is through interactions with unmethylated CpG islands [33]–[35]. It is therefore intriguing that SBSN, whose promoter contains no CpG islands, had high levels of H3K27me3 in undifferentiated keratinocytes; there is a broad region of H3K27me3 with a peak ∼800 bp upstream from the TSS (Figure S7B). Taking advantage of the publically available ENCODE histone modification ChIP-seq data we studied globally the overlap of H3K27me3 with CpG islands and their 500 bp flanking regions. While in ES cells 43 percent of H3K27me3 regions did overlap with CpG islands, only 17 percent of H3K27me3 regions in undifferentiated NHEKs overlapped with CpG islands. We also carried out this analysis on H3K27me3 regions in Normal Human Lung Fibroblasts and found they also had a 17 percent overlap with CpG islands. This finding supports the hypothesis that in more differentiated cells types such as epidermal keratinocytes and lung fibroblasts, PRC recruitment and H3K27me3 are largely mediated by mechanisms unrelated to unmethylated CpG islands. A second group, *BLNK* and *IVL*, displayed much less change in H3K27me3, with a more prominent increase in H3K4me3 (Figure 6B, Figure S7C). These two groups are likely regulated by a combinatorial PcG/trxG mechanism. In contrast a third group of differentiation genes, including *TGM1* and *SCEL* show nearly no H3K27me3 in progenitor or differentiated cells, but display a dramatic increase in H3K4me3 upon differentiation (Figure 6C, Figure S7D). These findings support a model where activation of differentiation genes in epidermal progenitors is either mediated through a joint, reciprocal PcG/trxG coregulation, or by trxG alone (Figure 6D).

## Discussion

Our study presents three major findings significant for understanding the control of epithelial cell differentiation. First we defined a role for Trithorax proteins WDR5 and MLL2 in activating the differentiation gene program in epidermal progenitor cells. Through siRNA knock down experiments, whole genome gene expression microarrays and ChIP experiments, we demonstrate that the histone methyltransferase, MLL2, activates many genes involved in different stages of epidermal progenitor cell differentiation. We also discovered, through ChIP-sequencing experiments, that a core component of the histone methyltransferase complex, WDR5, occupies promoters of a large subset of genes involved in epidermal progenitor differentiation. While previous work has demonstrated that WDR5 is involved in maintaining the ES cell state [36], our work shows that WDR5 is also important for promotion of terminal differentiation in the epidermal lineage. The most probable explanation for the diversity in WDR5 function is selective recruitment to the appropriate promoters by cell- and differentiation-specific DNA binding proteins. Thus, in ES cells trxG complexes are recruited to gene targets through direct interactions with Oct4, while in epidermal keratinocytes recruitment is through interactions with epidermal transcription factors such as GRHL3. The SET domain containing proteins in the trxG complex are the subunits that are enzymatically responsible for methyltransferase activity, therefore described as “writers”. These proteins include the MLL and SET proteins, the mammalian homologues to drosophila *trx*. The apparent non-redundant functions of the mammalian MLL proteins, as demonstrated by the embryonic lethality in mice upon deletion or truncation of individual family members [37]–[40], suggests that different MLL proteins may act as unique components in trxG complexes aiding in their gene specific recruitment. Identifying the specific SET domain containing MLL protein, MLL2, as having a role in promoting epidermal differentiation broadens our understanding of the role of MLL proteins in differentiation of a self-renewing tissue. We also observed MLL1 binding to some epidermal differentiation genes, suggesting that other MLLs and SET may play a role in activating the epidermal differentiation program. Recently mutations in *MLL2* have been found with high incidence rate in patients with Kabuki syndrome, a syndrome with multiple congenital abnormalities and intellectual disabilities [41]–[47]. No skin abnormalities have been described in Kabuki syndrome which may be because the syndrome is caused by haploinsufficiency [41], [47] and the predicted fifty percent reduction in MLL2 expression may not be sufficient to affect epidermal differentiation *in vivo*.

Secondly we define a previously unknown role for the transcription factor GRHL3 in the recruitment of a trxG complex to promoters of genes, leading to increased H3K4 methylation and gene expression. The critical role for Grhl3 in epidermal differentiation was previously shown in the mouse [9], [10], and we now demonstrate a similar role in activation of the human epidermal differentiation gene expression program. Our genome wide gene expression analysis on cells depleted of either GRHL3 or MLL2 revealed 109 genes co-regulated by these two factors, most of which have an increase in expression late in epidermal progenitor cell differentiation. Furthermore, we determined that GRHL3 directly interacts with WDR5 in differentiated keratinocytes and through ChIP-sequencing experiments we established a global co-localization of these two factors, with a significant enrichment of occupancy at genes involved in processes crucial for proper epidermal progenitor differentiation. Thus, we hypothesize that GRHL3 recruits trxG to epidermal differentiation promoters through WDR5. While the expression of MLL2 and other trxG components do not change during epidermal differentiation, the expression of GRHL3 does increase, possibly explaining how trxG is directed to its differentiation associated gene targets in a differentiation dependent manner. As GRHL3 plays roles in the differentiation of other epithelia, including bladder epithelium [48], we speculate that trxG may be similarly involved in activation of other epithelial differentiation programs.

Lastly we propose models of trxG mediated regulation of differentiation that are dependent and independent of functional interactions with PcG. While previous studies demonstrated roles for the PcG complex in maintaining the progenitor state [16], and demethylation of H3K27 for de-repression of epidermal differentiation genes [19], the current work suggests that this mechanism alone cannot fully explain activation of the differentiation program. This finding is consistent with the aforementioned studies as they found that only a subset of differentiation associated genes are affected upon disruption of either Ezh2 or JMJD3 [16], [19]. Activation of differentiation genes that are suppressed by PcG in progenitor cells appears to be associated with recruitment of trxG and increased H3K4me3 at the gene promoters during the differentiation process. This mode of regulation for differentiation genes is reminiscent of the antagonistic actions of trxG and PcG in fate determination in *Drosophila* [49]. However, the promoters of a subset of differentiation genes, including *TGM1*, whose activation is associated with H3K4me3 are not marked by H3K27me3 in the progenitor state; these genes primarily rely on a trxG mediated mechanism for activation and their low expression in progenitor cells does not appear to be associated with PcG mediated H3K27me3. These findings are consistent with *in vitro* studies where trxG could activate transcription on a chromatin template without the presence of PcG proteins [50] and where during induced gene activation, MLL2 can associate transiently with the Myc locus [51]. However, it should be pointed out that the set of epidermal differentiation genes that we find to be independent of regulation by PcG in the NHEK differentiation model may have been regulated by PcG earlier in their lineage similar to that observed for GATA3 gene activation during T cell development [52].

In summary, our work supports a previously unappreciated role for trxG in promoting expression of the human epidermal progenitor differentiation program; reveals the role of a transcription factor GRHL3 in recruiting this complex to its gene targets; and uncovers a function for trxG mediated gene activation in differentiation that is independent of overcoming PcG mediated repression.

## Materials and Methods

### Cell culture

Neonatal Human Epidermal Keratinocytes (NHEK) were purchased from LifeLine Technologies and grown according to the manufacturer's instructions in Dermalife medium (LifeLine Tech) supplemented with Dermalife growth factors (LifeLine Tech). For ChIP-qPCR experiments, cells were grown for two days and for ChIP-seq experiments cells were grown for 24 hours in medium supplemented with a final concentration of 1.8 mM CaCl_2_ to induce differentiation. For timecourse experiment, cells were seeded into a 6 well plate and induced to differentiate at 50% confluency by addition of 1.8 mM CaCl_2_ and collected at 0,1,3,6,12,24,and 48 hours after induction. RT4 cell were maintained in McCoy's 5A medium (Gibco) supplemented with 10% FBS. 293T cells were grown in DMEM (Gibco) supplemented with 10% FBS.

### Transfections

For siRNA experiments, NHEK cells were subjected to reverse transfection using Lipofectamine RNAi max (Life Tech Inc.) per manufacturer's protocol. 15 hours post transfection, medium was changed to Dermalife supplemented with 1.8 mM CaCl_2_ and cells were allowed to grow for 48 hours. The following Silencer Select siRNAs (Ambion) were used at a final concentration of 25 nM: GRHL3 (cat#s33754) MLL1 (cat#s8819) MLL2 (cat#s15604) MLL4 (cat #s18831) AshL1 (cat #s31702) WDR5 (cat #s225470) Negative #1 (cat#4390843). For HA-GRHL3 overexpression, plasmid was transfected into 293T cells with Lipofectamine 2000 (Life Tech Inc.) per manufacture's protocol.

### Proliferation, TUNEL, and senescence assays

Proliferation assays were performed by quantifying total ATP content via the ApoSENSOR ATP Luminescence luciferase reporter assay (cat# K254-1000, Bio Vision Inc.). TUNEL assays were performed using In Situ Cell Death Detection Kit, Fluroscein (cat# 11 684 795 910, Roche). Senescence assays were performed using Senescence β-Galactosidase Staining Kit (cat# 9860, Cell Signaling Technology, Inc.).

### Immunofluorescence

Immunofluorescence was performed as previously described [53] using 4% PFA fixed tissue. The following antibodies were used: MLL1 (Thermo Scientific, cat#PA5-11264, 1∶100), MLL2 (Abcam, cat#AB32474, 1∶100), WDR5 (R&D Systems, cat#AF5810, 1∶100), K5 (Covance, 1∶1000), K10 (Covance, 1∶1000), p63 (Santa Cruz, sc-8431, 1∶200) AlexaFluor anti-Rabbit 488 (Invitrogen, cat#A11008, 1∶500), AlexaFluor anti-Goat (Invitrogen, cat#A11078, 1∶500) and AlexaFluor anti-mouse 594 (Invitrogen, cat#A11005, 1∶500).

### RNA extractions

Cells were collected and lysed in Trizol, followed by Chloroform extraction. RNA was extracted from the aqueous phase using Ambion PureLink RNA mini kit per manufacturer's protocol. RNA concentration and quality were quantified on a NanoDrop.

### Microarray analysis

All experiments were performed with biological duplicates. Experiments were performed as previously described [54] except Affymetrix Human Gene 1.0 ST arrays (26,869 probe sets) were used and washed according to manufacturer's recommendations (Affymetrix, Santa Clara, CA). For the time course ANOVA was performed, using MeV software [55], [56] to analyze genes for differential expression. K-means clustering was performed on the differentially expressed genes as determined by ANOVA with a p<0.005 and greater than +/−1.5 fold change. siRNA microarrays were analyzed with Cyber-T [57]. The Neg siRNA was used as the control and either Grhl3 siRNA-treated or Mll2 siRNA-treated samples as experimental. Gene Ontology analysis was performed on all datasets using DAVID [58], [59]. Statistical significance of overlaps was calculated using the Fisher's exact test. The microarray data discussed in this publication have been deposited in NCBI's Gene Expression Omnibus [60] and are accessible through GEO series accession numbers GSE37570, GSE37049, and GSE38628.

### Chromatin immunoprecipitation assays

ChIP assays were performed as previously described [48] with the following changes: 24 ug of sonicated chromatin was used for each IP and enrichment was calculated as a percent of input sample compared to an IgG control IP and normalized to a control genomic region (n≥3). The following antibodies were used : Grhl3 (Andersen Lab) MLL2 (AbCam cat# ab32474) MLL1 (Bethyl cat#A300-374A) SETD1A (Abcam cat# ab70378) IgG (Sigma cat#15006-10MG) H3K4me1 (AbCam cat# ab8895) H3K4me2 (AbCam cat# ab32356) H3K4me3 (Milipore cat#07-473) H3K27me3 (AbCam cat#ab6002) WDR5 (AbCam cat#ab56919)

Primer sequences available upon request.

### Quantitative real-time PCR

For mRNA expression analysis cDNA was prepared using iScript cDNA kit (Biorad Laboritories) and RT-PCR was performed using SsoFast for Probes and SsoFast EvaGreen (Biorad Laboratories) master mixes in CFX384 Real-Time PCR Detection System (Biorad Laboratories). GAPDH or RPLPO were used as endogenous controls. RT-PCR was performed using the following primers or probes (n≥3): Taqman Probes: WDR5: Hs00424605_m1 TGM1: Hs00165929_m1

Krt10: Hs00166289_m1 Grhl3: Hs00297962_m1 Grhl1: Hs00227745_m1 Ppl: Hs00160312_m1

Primers: Brip1 Fwd: TTACCCGTCACAGCTTGCTAT Rv: TCCCACTAAGAGATTGTTGCCA Ets1 Fwd: AGACGGAAAAAGTCGATCTGGA Rv: TGCTTGGAGTTAATAGTGGGACA

Fos Fwd: CGGGCTTCAACGCAGACTA Rv: GGTCCGTGCAGAAGTCCTG

Jun Fwd: TGGAAACGACCTTCTATGACGA Rv: GTTGCTGGACTGGATTATCAGG

Klf4 Fwd: GCGCTGCTCCCATCTTTCT Rv: TGCTTGACGCAGTGTCTTCTC

Creb5 Fwd: CCCTGCCCAACCCTACAATG Rv: GGACCTTGCATCCCCATGAT

### Co-immunoprecipitation

NHEK cells were differentiated for two days prior to cell collection. 293T cells were collected 2 days post transfection with HA-GRHL3. Cells were lysed in 1%NP-40 lysis buffer on ice for 1 hour with vortexing every 5 minutes. Protein extract was pre-blocked with protein A- agarose beads (Invitrogen) for 45 minutes at 4C with constant rotation. Protein extract was incubated with 5 ug of indicated primary antibodies overnight at 4C: IgG (Sigma), WDR5 (Abcam), MLL1 (Bethyl), MLL2 (Abcam), SETD1A (Abcam), HA (Covance), and Grhl3 (Andersen Lab). Samples were immunoprecipitated using pre-blocked protein A Dynabeads (Invitrogen) for 1 hour at 4C, followed by washing in PBS and elution in loading buffer at 100C for 10 minutes.

### Western blot

Protein samples were run on a 4–20% gradient gel (Invitrogen) and transferred to a PVDF membrane. The membrane was blocked in 5% milk, washed with 1xPBS-T and incubated in 1% milk with indicated antibodies: Grhl3 (Andersen Lab), MLL2 (AbCam), MLL1 (Bethyl) , SETD1A (AbCam), WDR5 (AbCam), HA (Covance) followed by incubation in secondary antibodies anti-rabbit HRP or anti-mouse HRP. Signal was detected using ECL per manufacturer's protocol (Denville).

### ChIP–Seq

Sequencing libraries were generated for the GRHL3, WDR5, and Input samples using the NEB Next reagents and Illumina adaptors and oligos, according to the Illumina protocol for ChIP-Seq library preparation, with some modification. After adaptor ligation, PCR amplification was performed prior to size selection of the library [61]. Clustering and 50-cycle single end sequencing were performed on the Illumina Hi-Seq 2000 Genome Analyzer. Resulting reads were aligned using Bowtie [62], and only uniquely aligning reads were retained. Peaks were called using MACS [63], and Galaxy [64]–[66] was used for further analysis.

## Supporting Information

Figure S1 GRHL3 regulation of *TGM1*. (A) qRT-PCR of *GRHL3* expression in human bladder epithelia cells (RT4), undifferentiated normal human epidermal keratinocytes (NHEK U), and differentiated normal human epidermal keratinocytes (NHEK D). (B) *GRHL3* and *TGM1* mRNA levels upon knockdown of GRHL3 (GRHL3 siRNA) compared to scrambled siRNA control (Neg siRNA).
(TIF)

Figure S2 Expression of Trithorax group members in human and mouse skin. (A) Expression of Trithorax family members during calcium-induced human epidermal keratinocyte differentiation. (B) qRT-PCR of GRHL3, WDR5, MLL2, and MLL1 in human whole skin. (C) qRT-PCR of Grhl3, Wdr5, Mll2, and Mll1 in mouse skin separated into dermal and epidermal samples. (D–H) Immunofluroescence in normal human skin. (I) qRT-PCR of MLL1, MLL2 and WDR5 upon knockdown of indicated genes by siRNA.
(TIF)

Figure S3 Gene expression during human epidermal keratinocyte differentiation. (A) Flow chart of microarray analysis. (B) Gene Ontology (GO) analysis of significantly changing genes during the keratinocyte differentiation timecourse.
(TIF)

Figure S4 Validation of human epidermal keratinocyte differentiation microarray. Blue graphs are representative genes in the early cluster. Green graphs are representative genes from the intermediate cluster. Peach graphs are representative genes in the late cluster.
(TIF)

Figure S5 GRHL3 and MLL2 in human epidermal keratinocyte differentiation. (A–B) Flow chart for microarray data analysis of GRHL3 (A) and MLL2 (B) siRNA depleted NHEK cells. (C) GRHL3 binding site analysis in promoters of genes downregulated upon GRHL3 and MLL2 depletion. (D–E) Significance for the overlap of genes belonging to differentiation clusters and genes downregulated by GRHL3 siRNA (D) and genes downregulated by MLL2 siRNA (E). p values in (D–E) calculated by Fisher's exact test.
(TIF)

Figure S6 WDR5 and GRHL3 co-localize to regulate human epidermal keratinocyte differentiation. (A–B) Co-Immunoprecipitation in HA-GRHL3 transfected 293T cells. Cell extracts were immunoprecipitated with the indicated antibodies and blots were probed with the indicated antibodies (IgG was used as a control). * denotes predicted band size. (C) Number of peaks in GRHL3 and WDR5 ChIP-seq experiment. (D) Distribution of WDR5 and GRHL3 ChIP-sequencing peaks in promoter, intergenic and intragenic regions compared to the average distribution of these regions. (E) Percentage of differentially expressed genes in human keratinocyte differentiation timecourse described in Figure 1B that are co-occupied by WDR5 and GRHL3. (F) Percent of genes in each previously defined differentiation cluster bound by WDR5, GRHL3 or both factors.
(TIF)

Figure S7 PcG and trxG regulation in epidermal differentiation. (A) ChIP assay with H3K27me3 and H3K4me3 antibodies 1 and 3 hours post calcium-induced differentiation in NHEK D cells. (B) ChIP assay with H3K27me3 antibodies 1 and 3 hours post calcium-induced differentiation in NHEK D cells. (C–D) ChIP assay with H3K27me3 and H3K4me3 antibodies 1 and 3 hours post calcium-induced differentiation in NHEK D cells.
(TIF)

Table S1 Genes with differential gene expression during calcium induced NHEK differentiation.
(XLSX)

Table S2 Genes that are repressed or induced with GRHL3 knockdown.
(XLSX)

Table S3 Genes that are repressed or induced with MLL2 knockdown.
(XLSX)
