# Supplementary figures and images for: GRHL3/GET1 and Trithorax Group Members Collaborate to Activate the Epidermal Progenitor Differentiation Program
Source: PLoS Genet. 2012 Jul 19;8(7):e1002829. doi: 10.1371/journal.pgen.1002829 (PMC3400561; doi:10.1371/journal.pgen.1002829)

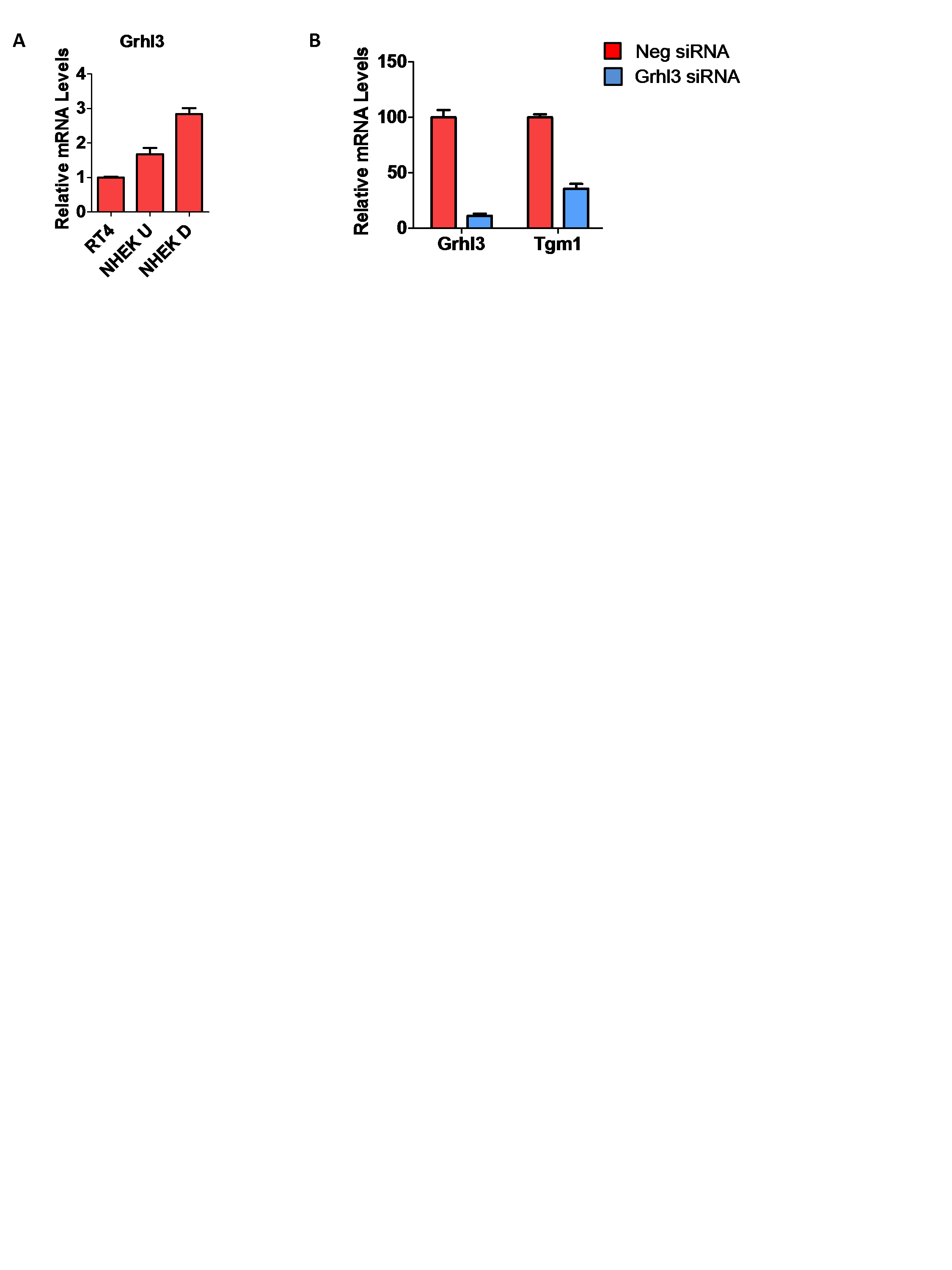

Supplement: Figure S1 — GRHL3 regulation of TGM1. (A) qRT-PCR of GRHL3 expression in human bladder epithelia cells (RT4), undifferentiated normal human epidermal keratinocytes (NHEK U), and differentiated normal human epidermal keratinocytes (NHEK D). (B) GRHL3 and TGM1 mRNA levels upon knockdown of GRHL3 (GRHL3 siRNA) compared to scrambled siRNA control (Neg siRNA). (TIF) [file pgen.1002829.s001.tif]

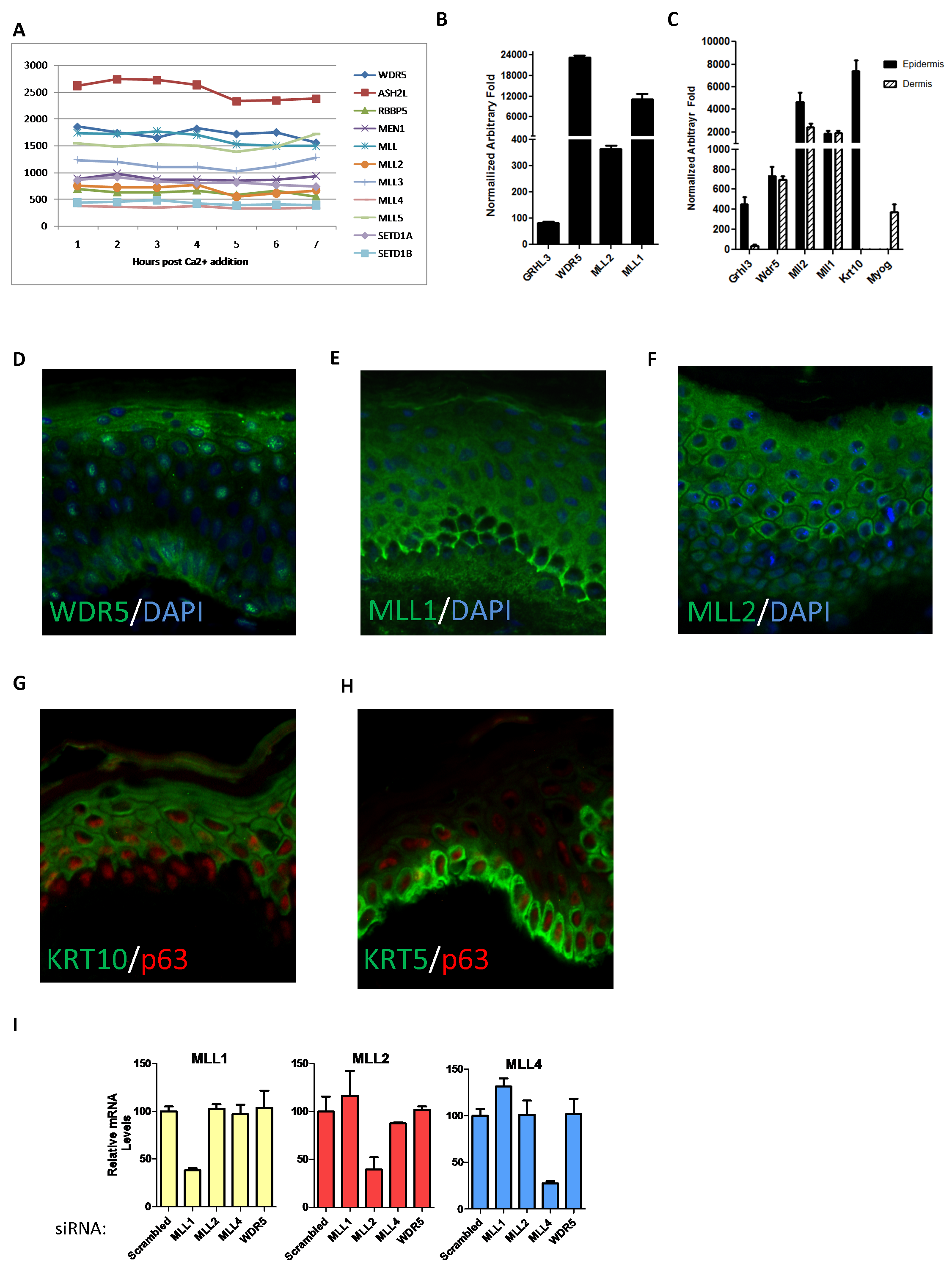

Supplement: Figure S2 — Expression of Trithorax group members in human and mouse skin. (A) Expression of Trithorax family members during calcium-induced human epidermal keratinocyte differentiation. (B) qRT-PCR of GRHL3, WDR5, MLL2, and MLL1 in human whole skin. (C) qRT-PCR of Grhl3, Wdr5, Mll2, and Mll1 in mouse skin separated into dermal and epidermal samples. (D–H) Immunofluroescence in normal human skin. (I) qRT-PCR of MLL1, MLL2 and WDR5 upon knockdown of indicated genes by siRNA. (TIF) [file pgen.1002829.s002.tif]

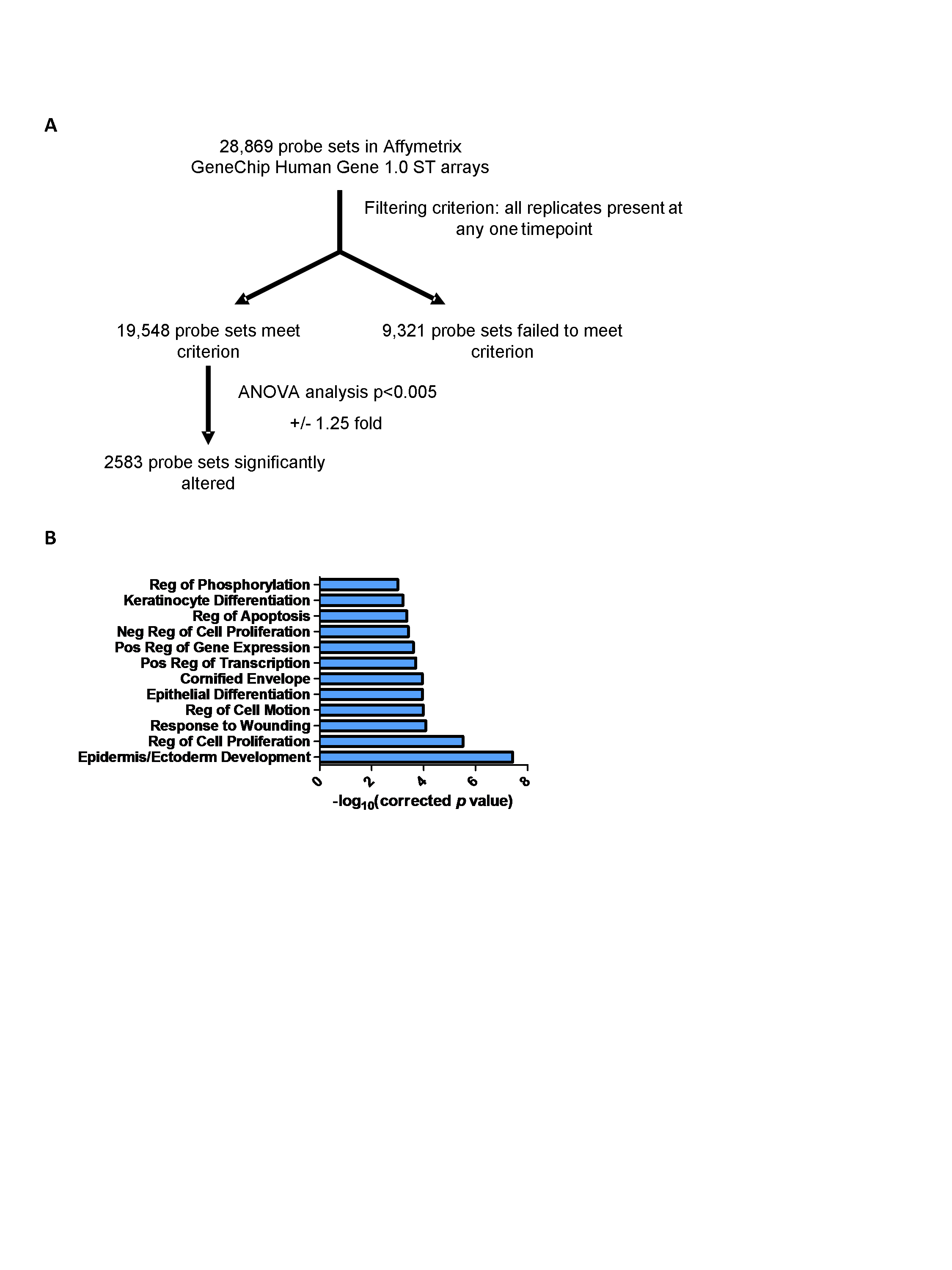

Supplement: Figure S3 — Gene expression during human epidermal keratinocyte differentiation. (A) Flow chart of microarray analysis. (B) Gene Ontology (GO) analysis of significantly changing genes during the keratinocyte differentiation timecourse. (TIF) [file pgen.1002829.s003.tif]

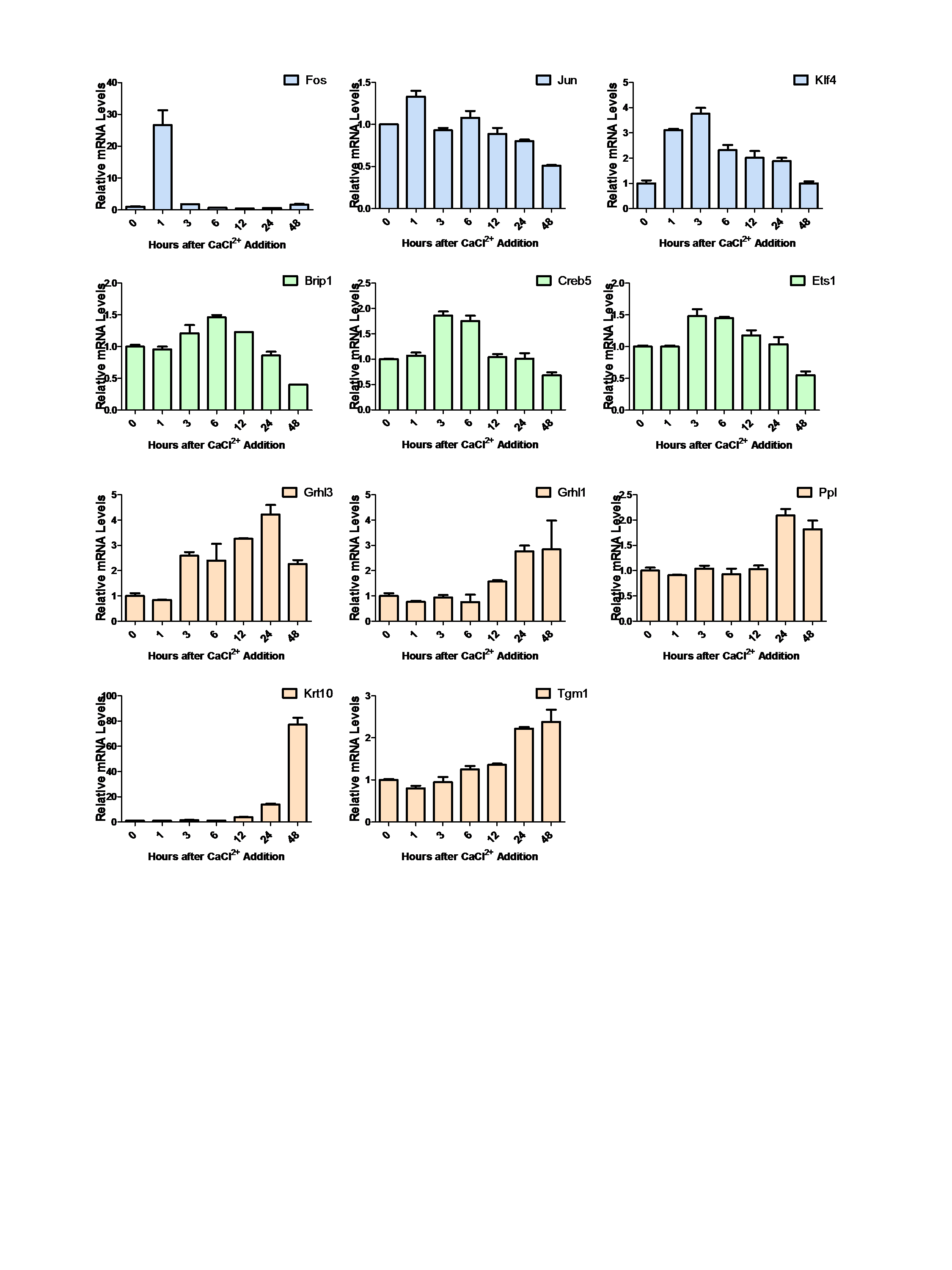

Supplement: Figure S4 — Validation of human epidermal keratinocyte differentiation microarray. Blue graphs are representative genes in the early cluster. Green graphs are representative genes from the intermediate cluster. Peach graphs are representative genes in the late cluster. (TIF) [file pgen.1002829.s004.tif]

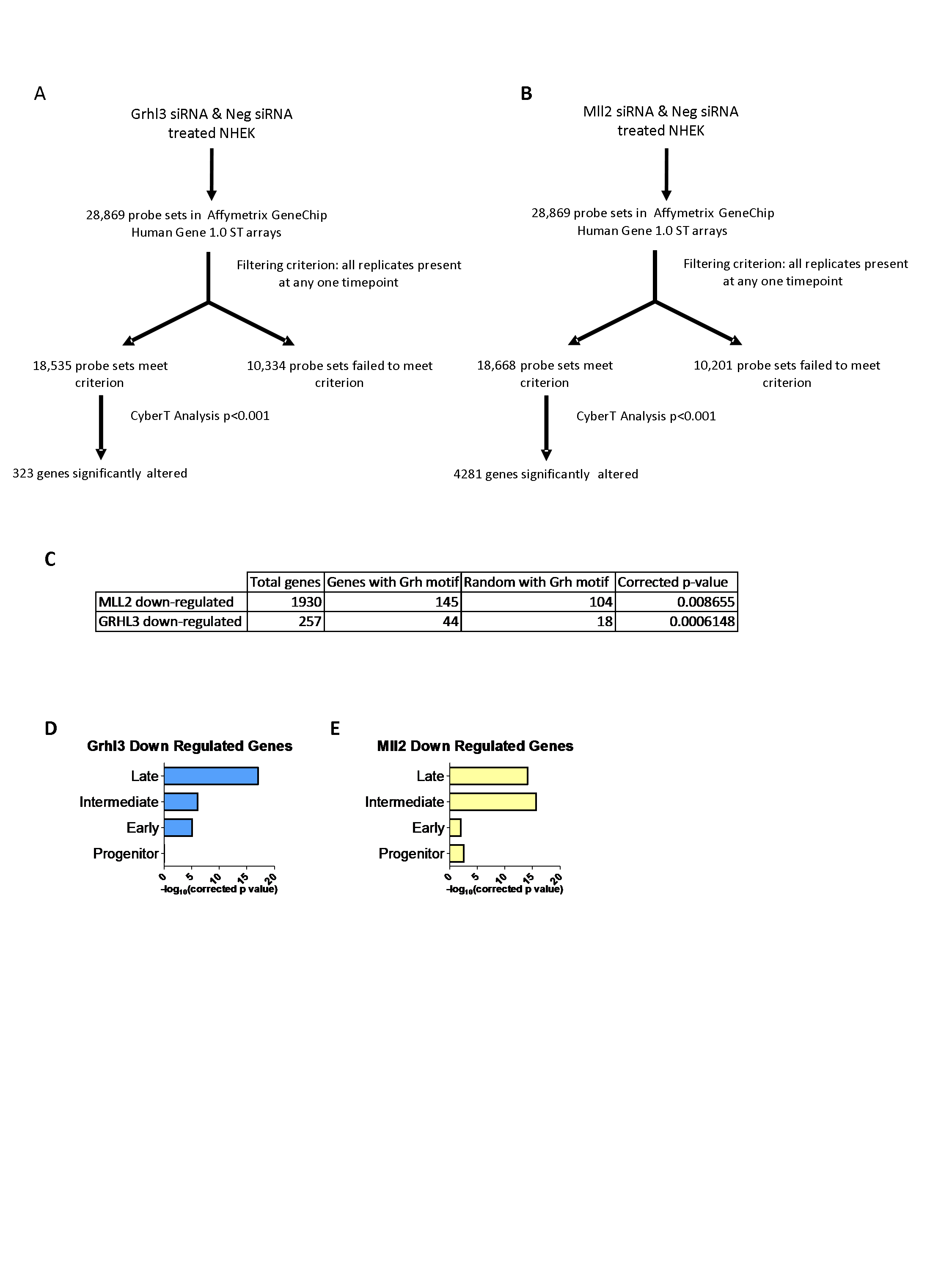

Supplement: Figure S5 — GRHL3 and MLL2 in human epidermal keratinocyte differentiation. (A–B) Flow chart for microarray data analysis of GRHL3 (A) and MLL2 (B) siRNA depleted NHEK cells. (C) GRHL3 binding site analysis in promoters of genes downregulated upon GRHL3 and MLL2 depletion. (D–E) Significance for the overlap of genes belonging to differentiation clusters and genes downregulated by GRHL3 siRNA (D) and genes downregulated by MLL2 siRNA (E). p values in (D–E) calculated by Fisher's exact test. (TIF) [file pgen.1002829.s005.tif]

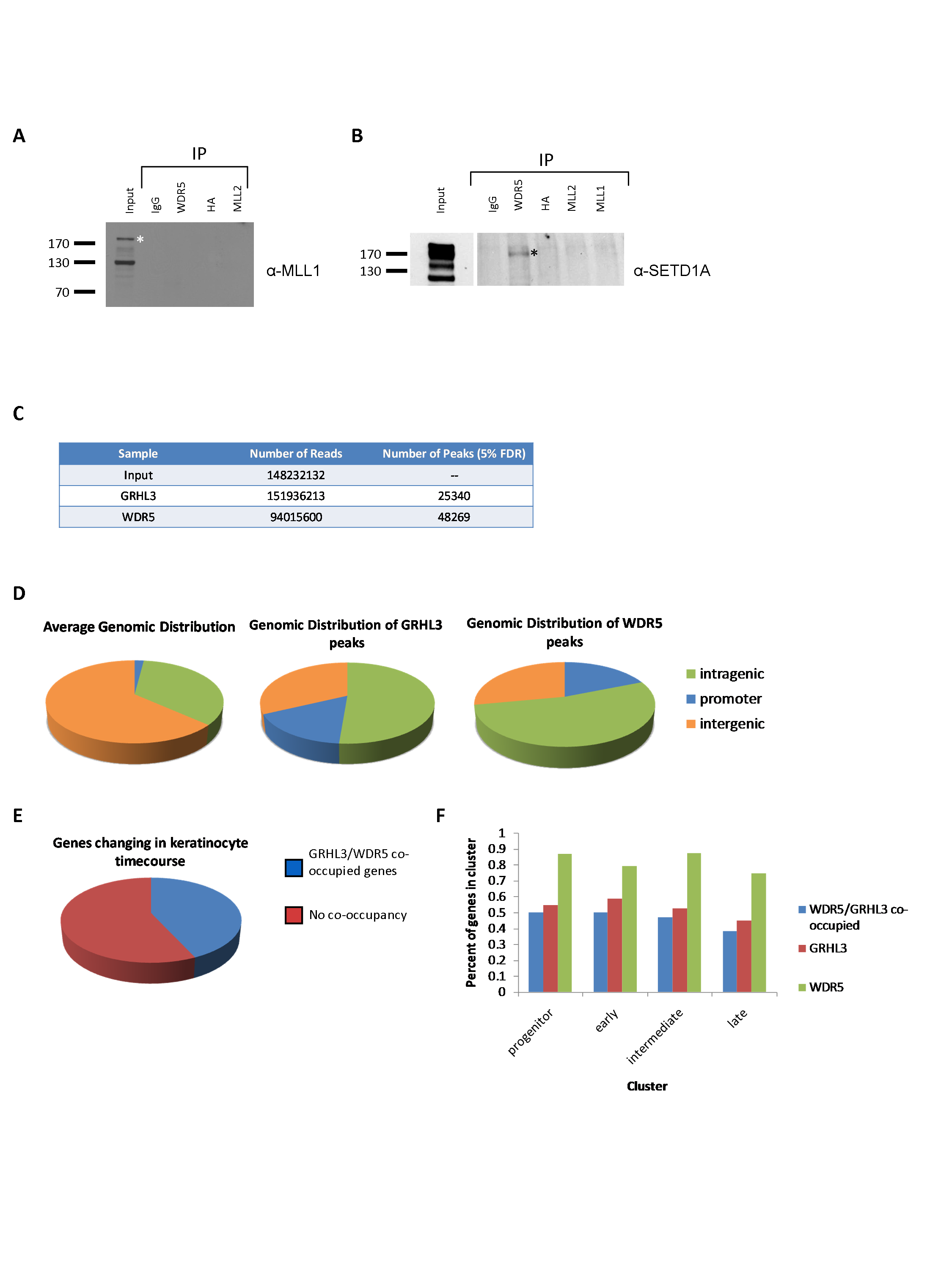

Supplement: Figure S6 — WDR5 and GRHL3 co-localize to regulate human epidermal keratinocyte differentiation. (A–B) Co-Immunoprecipitation in HA-GRHL3 transfected 293T cells. Cell extracts were immunoprecipitated with the indicated antibodies and blots were probed with the indicated antibodies (IgG was used as a control). * denotes predicted band size. (C) Number of peaks in GRHL3 and WDR5 ChIP-seq experiment. (D) Distribution of WDR5 and GRHL3 ChIP-sequencing peaks in promoter, intergenic and intragenic regions compared to the average distribution of these regions. (E) Percentage of differentially expressed genes in human keratinocyte differentiation timecourse described in Figure 1B that are co-occupied by WDR5 and GRHL3. (F) Percent of genes in each previously defined differentiation cluster bound by WDR5, GRHL3 or both factors. (TIF) [file pgen.1002829.s006.tif]

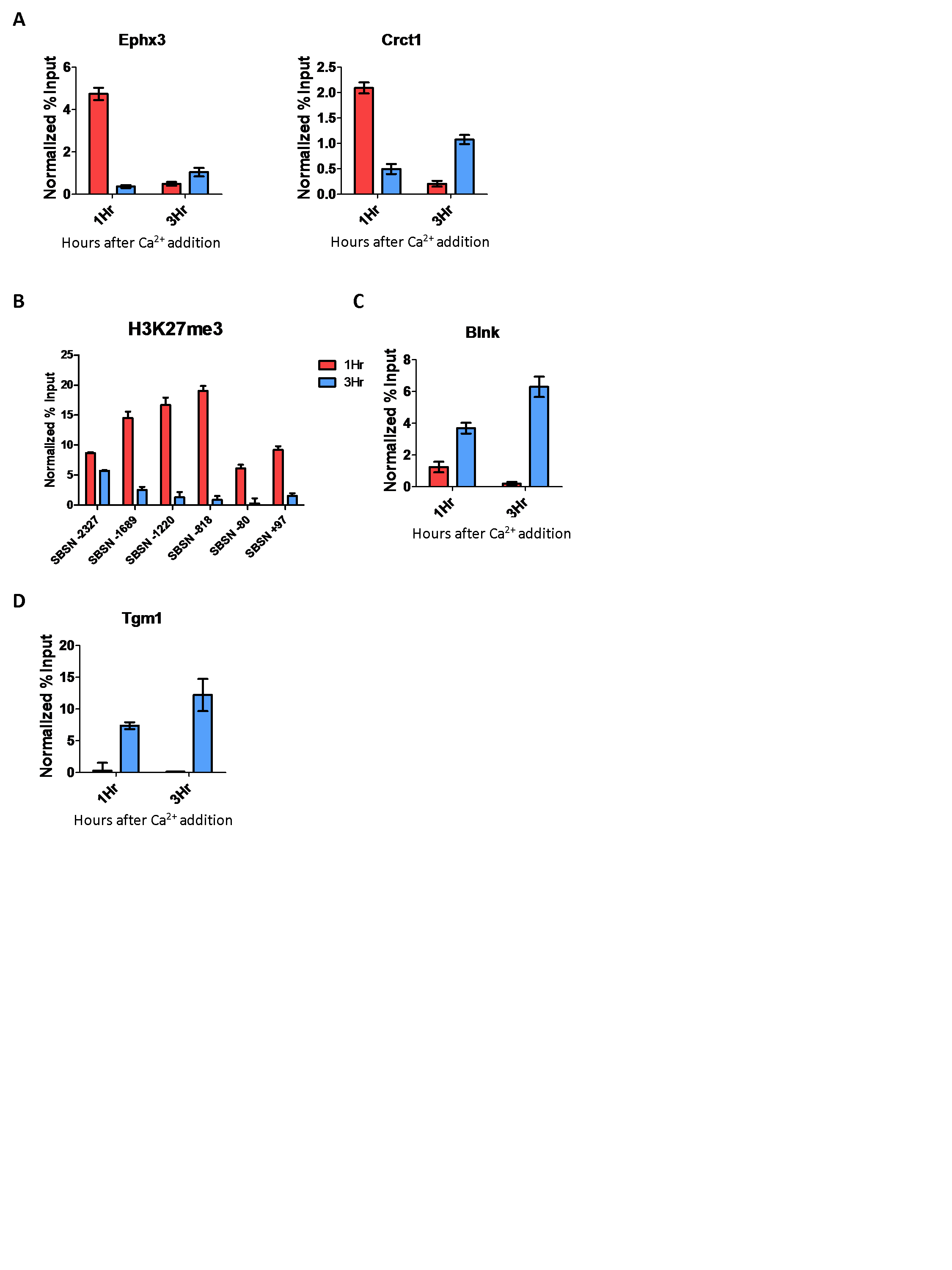

Supplement: Figure S7 — PcG and trxG regulation in epidermal differentiation. (A) ChIP assay with H3K27me3 and H3K4me3 antibodies 1 and 3 hours post calcium-induced differentiation in NHEK D cells. (B) ChIP assay with H3K27me3 antibodies 1 and 3 hours post calcium-induced differentiation in NHEK D cells. (C–D) ChIP assay with H3K27me3 and H3K4me3 antibodies 1 and 3 hours post calcium-induced differentiation in NHEK D cells. (TIF) [file pgen.1002829.s007.tif]
